# Effectiveness of Immediate Oral Calcium Supplementation After Thyroidectomy on Early Postoperative Hypocalcemia: A Retrospective Study

**DOI:** 10.7759/cureus.107612

**Published:** 2026-04-23

**Authors:** Gayatri Mini, Bryan Joel Devaraj Dhinahar, Nitin K B, Dhinahar Sundararaj

**Affiliations:** 1 General Surgery, JSS Academy of Higher Education and Research, Mysore, IND; 2 Internal Medicine, Sheffield Teaching Hospital NHS Foundation trust, Sheffield, GBR; 3 General Surgery, King George Hospital, London, GBR; 4 Orthodontics and Dentofacial Orthopaedics, Rose Medical Centre, Chennai, IND

**Keywords:** hypocalcaemia, oral calcium, postoperative care, prophylaxis, retrospective cohort, thyroidectomy

## Abstract

Background: Hypocalcemia is the most frequent complication following total thyroidectomy (TT), associated with neuromuscular symptoms and prolonged hospitalization. Although prophylactic oral calcium supplementation is commonly used to prevent postoperative hypocalcemia, its routine use remains debated.

Objective: This study aimed to assess the effectiveness of immediate postoperative oral calcium supplementation in reducing the incidence of hypocalcemia following thyroidectomy.

Methods: This single-center retrospective cohort study included patients who underwent TT or partial thyroidectomy (PT) between January 2018 and December 2022. Patients were categorized into two groups: those who received immediate postoperative oral calcium prophylaxis (most commonly calcium carbonate 500-1000 mg daily, with or without vitamin D, based on surgeon preference) and those who received standard care without prophylaxis. The primary outcome was hypocalcemia within 48 hours, defined as total serum calcium <8.5 mg/dL and/or the presence of clinical symptoms. Categorical variables were compared using the chi-square test, and statistical significance was set at p < 0.05.

Results: Seventy-four patients were initially screened; 72 patients (mean age: 44.9 ± 12 years; 82.4% female) were included in the final analysis after excluding two with missing postoperative data. Among them, 21 received calcium prophylaxis and 51 received standard care. Hypocalcemia occurred in 0% of patients in the prophylaxis group compared to 35.3% in the control group (p = 0.002). Stratified analysis of the high-risk TT group (n=52) showed a significant reduction in hypocalcemia from 54.5% (18/33) in the control group to 0% (0/19) in the prophylaxis group (p < 0.001). No adverse events related to calcium supplementation were observed.

Conclusions: Immediate postoperative oral calcium supplementation significantly reduces early postoperative hypocalcemia after thyroidectomy. Given its safety, low cost, and clinical effectiveness, routine calcium prophylaxis should be considered as part of standardized postoperative care. Further prospective multicenter trials are warranted to validate these findings.

## Introduction

Postoperative hypocalcemia is the most common metabolic complication following thyroid surgery and remains a significant cause of postoperative morbidity [1]. The risk of hypocalcemia, however, is not uniform across all thyroid procedures. It is substantially higher after a total thyroidectomy (TT) compared to a partial thyroidectomy (PT) or hemithyroidectomy, owing to greater parathyroid gland manipulation, potential devascularization, and altered postoperative parathyroid hormone (PTH) dynamics.

Routine postoperative oral calcium supplementation is widely used to prevent early hypocalcemia; however, its universal application remains controversial. Quantitative studies have reported transient hypocalcemia rates ranging from 20% to 50% following TT, with a significantly lower incidence observed among patients receiving routine calcium supplementation [2,3]. While some centers advocate selective supplementation guided by early postoperative biochemical markers such as PTH, the practicality of this approach is limited in many settings by the availability and turnaround time of such assays [4].

Evidence evaluating pragmatic calcium prophylaxis strategies in real-world clinical practice, particularly from Indian tertiary care centers and across commonly performed thyroidectomy procedures, remains limited [5]. This study aimed to assess the effectiveness of immediate postoperative oral calcium supplementation in reducing early postoperative hypocalcemia following thyroidectomy by comparing outcomes between patients who received routine calcium prophylaxis and those who did not, with consideration of the extent of thyroid resection.

## Materials and methods

Materials and methods

Study Design and Setting

This study employed a retrospective cohort design using a convenience sampling technique at JSS Hospital, a tertiary care center in Mysore, India. The study population was identified through a systematic review of the hospital’s electronic and physical medical records for all patients who underwent thyroid surgery between January 2018 and December 2022.

To enhance scientific rigor and address potential confounding factors related to the extent of surgery, as the risk of hypocalcemia is not uniform across different procedures, patients were stratified into two primary cohorts: TT, including TT with or without neck dissection, and PT, including hemithyroidectomy and completion thyroidectomy. This stratification allowed differentiation between patients undergoing complete gland removal and those undergoing partial resection, which carry differing risks of postoperative parathyroid dysfunction.

Inclusion and Exclusion Criteria

Inclusion criteria: Adult patients (≥18 years) who underwent either TT or PT during the study period and had available postoperative biochemical data (serum calcium and PTH, where available) within 48 hours of surgery.

Exclusion criteria: Patients with pre-existing parathyroid disease, chronic kidney disease stage IV or V, preoperative hypocalcemia, documented vitamin D deficiency, or prior reoperative neck surgery (excluding completion thyroidectomy) were excluded.

Definition of Outcome

Postoperative hypocalcemia was defined as total serum calcium <8.5 mg/dL (reference range 8.5-10.5 mg/dL) within 48 hours of surgery and/or the presence of clinical signs or symptoms of hypocalcemia. Clinical manifestations assessed included perioral or fingertip paraesthesia, carpopedal spasm, muscle cramps, tetany, positive Chvostek’s sign, positive Trousseau’s sign, or prolonged QT interval on electrocardiogram (ECG).

Prophylactic regimen and postoperative care

Treatment Allocation

As no standardized institutional protocol for routine calcium prophylaxis existed during the study period, treatment decisions were made at the discretion of the operating consultant. Patients were categorized into two groups: the prophylaxis group (patients started on calcium supplementation irrespective of serum calcium levels) and the standard care group (patients monitored without routine prophylactic supplementation).

Intervention and Timing

Prophylactic supplementation was initiated postoperatively within 24-48 hours after surgery. No preoperative calcium loading was used. The supplementation regimen consisted of oral calcium carbonate providing approximately 500-1000 mg elemental calcium daily, often combined with calcitriol (0.25 mcg), and was typically continued for approximately 14 days.

Patients in the standard care group did not receive routine prophylactic supplementation. Calcium therapy was initiated only when clinically indicated based on biochemical or symptomatic hypocalcemia.

Route of Administration

Oral calcium was used for prophylactic supplementation. Intravenous calcium gluconate was reserved as rescue therapy at the consultant’s discretion for patients with symptomatic hypocalcemia, including tetany or carpopedal spasm, markedly reduced serum calcium levels (<7.5 mg/dL), or electrocardiographic evidence of QT interval prolongation.

Variables and data collection

Variables analyzed included age, gender, indication for surgery, type of thyroidectomy procedure, and serum calcium levels. Serum PTH levels were also reviewed, where available. However, as PTH was not routinely measured for all patients, particularly in PT cases, these values were used primarily to assess clinical trends rather than as a primary comparative variable.

Statistical analysis

Data were entered into Microsoft Excel (Microsoft Corp., Redmond, WA, USA) and analyzed using IBM SPSS Statistics software, version 22 (IBM Corp., Armonk, NY, USA). Continuous variables were expressed as mean ± standard deviation (SD), and categorical variables were presented as frequencies and percentages. Normality of continuous variables was assessed using the Shapiro-Wilk test. Between-group comparisons were performed using the chi-square (χ²) test or Fisher’s exact test for categorical variables, and the independent t-test or Mann-Whitney U test for continuous variables, as appropriate. A two-sided p-value <0.05 was considered statistically significant.

Sample size

All eligible patients during the study period were included (n = 74). As this was a retrospective analysis of a fixed cohort, no a priori sample size calculation was performed.

Ethical approval

This study was approved by the Institutional Ethics Committee of JSS Academy of Higher Education and Research (JSSAHER) in June 2022. A waiver of individual informed consent was granted, as only de-identified medical records were reviewed.

## Results

A total of 74 patients were included in the study. The majority were female (n = 61; 82.4%), with a mean age of 43.93 ± 12.04 years, while males (n = 13; 17.6%) had a mean age of 51.69 ± 12.04 years. Thus, thyroid disorders requiring surgical intervention were more common among women and tended to occur at a relatively younger age compared to men (Table 1).

**Table 1:** Distribution of surgical procedures performed in the study population

Surgical Procedures	Number of Patients (n)	Percentage (%)
Total Thyroidectomy	42	56.8
Hemithyroidectomy	16	21.6
Total Thyroidectomy With Neck Dissection	12	16.2
Hemithyroidectomy With Neck Dissection	1	1.4
Completion Thyroidectomy	3	4.1
Total	74	100

Regarding the type of surgery, TT was most frequent (56.8%), followed by hemithyroidectomy (21.6%) and TT with neck dissection (16.2%). Less common procedures included complete thyroidectomy (4.1%) and hemithyroidectomy with neck dissection (1.4%) (Table 1). This shows that complete gland removal was generally preferred, particularly in malignant or high-risk pathology.

The main indications for thyroidectomy are summarized in Table 2. Multinodular goiter (41.1%) was the leading diagnosis, followed by papillary carcinoma (26.0%), thyroid nodule (12.3%), follicular neoplasm (6.8%), and colloid goiter (5.5%). Less frequent but clinically relevant diagnoses included adenoma of thyroid (4.1%), Graves’ disease (1.4%), and rare atypical entities such as glottis carcinoma and follicular lesion of undetermined significance (FLUS) (1.4%).

**Table 2:** Distribution of diagnoses FLUS: follicular lesion of undetermined significance

Diagnosis	Number of Patients (n)	Percentage (%)
Multinodular Goitre	30	41.1
Follicular Neoplasm	5	6.8
Papillary Carcinoma of the Thyroid	19	26
Graves’ Disease	1	1.4
Carcinoma of the Glottis	1	1.4
Thyroid Nodule	9	12.3
Colloid Goitre	4	5.5
Adenoma of the Thyroid	3	4.1
FLUS	1	1.4
Total	73	100

One patient had missing diagnostic data (n = 73 used for percentage calculation). Postoperative hypocalcemia occurred in 18 of 72 patients (25%). Among those without calcium prophylaxis (n = 51), 18 (35.3%) developed hypocalcemia, whereas none (0%) of those receiving prophylaxis (n = 21) did. The difference was statistically significant (Pearson χ² = 9.882, df = 1, p = 0.002; Fisher’s exact p = 0.001) (Table 3). In the TT group (n=52), the incidence of hypocalcemia was 54.5% (18/33) in the standard care group compared to 0% (0/19) in the prophylaxis group, a difference that was highly statistically significant (p < 0.001). In the PT group (n=20), no episodes of biochemical or symptomatic hypocalcemia were observed in either the standard care (0/18) or prophylaxis (0/2) cohorts (Table 3). This confirms that while the baseline risk is predominantly concentrated in total resections where multi-gland manipulation is required, the prophylactic regimen remains 100% effective in preventing early postoperative calcium drops. In our study population, thyroid surgery was more commonly undertaken in middle-aged women, with multinodular goitre and papillary carcinoma as the leading indications. As TT was the predominant procedure, routine oral calcium prophylaxis was associated with a significant reduction in postoperative hypocalcemia [4].

**Table 3:** Relationship between postoperative calcium prophylaxis and hypocalcemia chi-square (χ²) = 9.882, df = 1, p = 0.002; Fisher’s exact = 0.001. Note: Two patients had missing postoperative calcium values; therefore, the hypocalcemia analysis was performed on 72 patients.

Postoperative Calcium Prophylaxis	Hypocalcemia Absent, n (%)	Hypocalcemia Present, n (%)	Total, n (%)
Not Given	33 (62.5%)	18 (37.7%)	51 (100%)
Given	21 (100%)	0 (0%)	21 (100%)
Total	54 (73%)	18 (27%)	72 (100%)

To address the varying risk profiles associated with the extent of surgery, we stratified our analysis into TT and PT cohorts. Biochemical markers in the TT group showed a mean postoperative calcium level of 8.28 ± 0.72 mg/dL and a mean PTH of 11.48 ± 17.32 pg/mL. In contrast, calcium levels remained higher in the PT group (8.43 ± 0.92 mg/dL), where PTH was not routinely measured due to the negligible risk of bilateral parathyroid damage (Table 4). 

**Table 4:** Stratified analysis of postoperative hypocalcemia by extent of thyroidectomy (total vs. partial) Postoperative hypocalcemia occurred in 18 of 72 patients (25%). To address potential confounding related to the extent of surgery, patients were stratified into total thyroidectomy and partial thyroidectomy groups.

Surgical Group	N	Postoperative Calcium (Mean ± SD)	Postoperative Parathyroid Hormone (Mean ± SD)	Hypocalcemia (Standard Care)	Hypocalcemia (Prophylaxis)
Total Thyroidectomy	52	8.28 ± 0.72	11.48 ± 17.32	18/33 (54.4%)	0/19 (0%)
Partial Thyroidectomy	20	8.43 ± 0.92	Not measured	0/18 (0%)	0/2 (0%)

## Discussion

The findings of this study demonstrate a significant reduction in early postoperative hypocalcemia among patients who received immediate oral calcium supplementation following thyroidectomy. An absolute risk reduction of 37.7% was observed, with no cases of hypocalcemia in the prophylaxis group compared with 35.3% in the non-supplemented group. This highlights the effectiveness of a simple, low-cost intervention in preventing one of the most common complications after thyroid surgery. A recent tertiary-care audit from eastern India similarly reported postoperative complication patterns consistent with our findings, supporting their broader applicability [5].

These results are consistent with previous studies evaluating the preventive role of calcium supplementation. Arer et al. [2], Jaan et al. [3], and Moore et al. [6] reported that routine calcium supplementation, either alone or in combination with vitamin D, significantly reduced the incidence of postoperative hypocalcemia. Earlier work assessing risk factors for post-thyroidectomy hypocalcemia has likewise demonstrated that proactive calcium replacement can mitigate biochemical and symptomatic disturbances [7]. Bellantone et al. [8] demonstrated that routine calcium supplementation, with or without vitamin D, is effective in reducing postoperative hypocalcaemia, supporting the role of early prophylactic therapy. Collectively, this evidence supports the rationale for early oral calcium administration to stabilize postoperative calcium levels and minimize symptomatic hypocalcemia.

The stratified analysis in our study further demonstrated that the protective effect of prophylactic calcium was particularly evident in patients undergoing total thyroidectomy, the group known to carry the highest risk of postoperative hypocalcemia.

Despite this, some authors favor a selective supplementation strategy based on early postoperative PTH measurement, which can identify high-risk patients while avoiding unnecessary treatment. However, rapid PTH testing is not consistently available in many centers, including ours, and delays in obtaining results may limit its usefulness in immediate postoperative decision-making, despite evidence supporting its predictive value for early postoperative hypocalcemia [8]. Under such circumstances, our findings provide pragmatic support for routine early supplementation as a safe and effective approach that may improve patient comfort and potentially reduce hospital stay.

These findings support the consideration of postoperative calcium prophylaxis as part of routine thyroidectomy care pathways, particularly in resource-limited settings where rapid biochemical predictors are not readily available.

Limitations

This single-center retrospective study has inherent limitations, including potential selection bias and variability in surgical technique. While a randomized controlled trial (RCT) would provide a higher level of evidence, this study offers valuable real-world clinical data. To address concerns regarding the inclusion of different surgical procedures, a stratified analysis focusing exclusively on the TT subgroup (n = 52) was performed. This targeted analysis confirmed that the prophylactic effect remained highly significant (p < 0.001) in the high-risk cohort in which all four parathyroid glands may be at risk, ensuring that the inclusion of partial resections did not dilute the overall findings.

Other limitations include a relatively modest sample size, a lack of uniform vitamin D assessment, and follow-up limited to the early postoperative period. These factors may limit generalizability; therefore, prospective multicenter trials with standardized protocols are recommended to further validate these findings.

## Conclusions

This study demonstrates that routine oral calcium supplementation, when initiated immediately after thyroidectomy, significantly reduces the incidence of early postoperative hypocalcemia. Stratified analysis suggests that the prophylactic effect is most clinically relevant in patients undergoing total thyroidectomy, where the risk of multi-gland manipulation is highest. Given its high safety profile, low cost, and clinical effectiveness, prophylactic calcium therapy should be incorporated into standard postoperative care pathways, particularly for TT, to improve surgical safety and patient outcomes. Further prospective, multicenter RCTs with standardized monitoring protocols are recommended to validate these findings and guide uniform postoperative management strategies.
